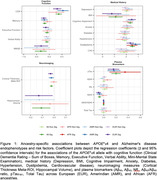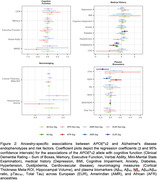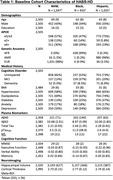# Ancestry‐specific effects of *APOE* on AD endophenotypes and modifiable risk factors

**DOI:** 10.1002/alz.095645

**Published:** 2025-01-09

**Authors:** Ana I Boeriu, Brian Fulton‐Howard, Nicole Phillips, Robert Barber, Sid E. O'Bryant, Kristine Yaffe, Shea J Andrews

**Affiliations:** ^1^ University of California, San Francisco, San Francisco, CA USA; ^2^ Icahn School of Medicine at Mount Sinai, New York, NY USA; ^3^ University of North Texas Health Science Center, Fort Worth, TX USA; ^4^ University of California, San Francisco and San Francisco VA Health Care System, San Francisco, CA USA

## Abstract

**Background:**

*APOE* is highly pleiotropic, with the e4 allele the strongest genetic risk factor for late‐onset Alzheimer’s disease (AD) and associated with other cardiometabolic traits. The APOE*e4 allele demonstrates ancestry‐specific differences in the risk for AD. Here, we investigated whether similar ancestry‐specific effects are observed across other AD endophenotypes and modifiable risk factors for AD.

**Method:**

Participants were 2505 non‐Hispanic Whites, Hispanics, and African Americans with genetic data from the Health and Aging Brain Study ‐ Health Disparities cohort (Table 1). Genetic ancestry was determined through principal component analysis, by projecting participants onto the 1000 Genomes reference panel. Global ancestry proportions were estimated using ADMIXTURE. The association of APOE*e4+ and *e2+ alleles with cognitive performance, plasma biomarkers, neuroimaging, and clinical comorbidities was investigated using regression models stratified by European (EUR), Amerindian (AMR), and African (AFR) ancestry. Covariates included age, gender, education; and intracranial volume for neuroimaging endophenotypes. Ancestry‐specific effects were determined by evaluating the equality in regression coefficients from the ancestry‐stratified models.

**Result:**

Genetic ancestry assignments were highly concordant with self‐reported race: 1071 EUR, 1003 AMR, and 431 AFR participants (Table 1). APOE*e4 was associated with lower BMI, poorer cognitive performance, increased risk of cognitive impairment and dyslipidemia, reduced cortical thickness and hippocampal volume, and plasma biomarkers levels ‐ higher pTau_181_, lower Ab_42_, and Ab_42_/Ab_40_ ratios (Figure 1). APOE*e2 was associated with reduced dyslipidemia risk (Figure 2). Notably, ancestry‐specific effects were observed, with APOE*e4 associated with lower Ab_42_/Ab_40_ in EUR compared to AMR. Additionally, APOE*e2 shows ancestry‐specific effects for Ab_42_/Ab_40_, pTau_181,_ and Ab_40_. In particular, AFR have a lower Ab_42_/Ab_40_ compared to both EUR and AMR. EUR have lower levels of pTau_181_ compared to both AFR and AMR. AFR have higher Ab_40_ compared to EUR and AMR.

**Conclusion:**

Our findings demonstrate that the impact of *APOE* on AD endophenotypes varies across ancestries, notably with amyloid and tau plasma biomarkers, and dyslipidemia. This underscores the critical need to consider genetic ancestry in assessing the effect of *APOE* on AD endophenotypes.